# Dimerization of the Sodium/Iodide Symporter

**DOI:** 10.1089/thy.2019.0034

**Published:** 2019-10-15

**Authors:** Rebecca J. Thompson, Alice Fletcher, Katie Brookes, Hannah Nieto, Mohammed M. Alshahrani, Jonathan W. Mueller, Nicholas H.F. Fine, David J. Hodson, Kristien Boelaert, Martin L. Read, Vicki E. Smith, Christopher J. McCabe

**Affiliations:** ^1^ Institute of Metabolism and Systems Research, University of Birmingham, Birmingham, United Kingdom.; ^2^ Centre for Endocrinology, Diabetes and Metabolism, Birmingham Health Partners, Birmingham, United Kingdom.

**Keywords:** NIS, radioiodide uptake, dimerization, thyroid

## Abstract

***Background:*** The ability of thyroid follicular epithelial cells to accumulate iodide via the sodium/iodide symporter (NIS) is exploited to successfully treat most thyroid cancers, although a subset of patients lose functional NIS activity and become unresponsive to radioiodide therapy, with poor clinical outcome. Our knowledge of NIS regulation remains limited, however. While numerous membrane proteins are functionally regulated via dimerization, there is little definitive evidence of NIS dimerization, and whether this might impact upon radioiodide uptake and treatment success is entirely unknown. We hypothesized that NIS dimerizes and that dimerization is a prerequisite for iodide uptake.

***Methods:*** Coimmunoprecipitation, proximity ligation, and Förster resonance energy transfer (FRET) assays were used to assess NIS:NIS interaction. To identify residues involved in dimerization, a homology model of NIS structure was built based on the crystal structure of the dimeric bacterial protein vSGLT.

***Results:*** Abundant cellular NIS dimerization was confirmed *in vitro* via three discrete methodologies. FRET and proximity ligation assays demonstrated that while NIS can exist as a dimer at the plasma membrane (PM), it is also apparent in other cellular compartments. Homology modeling revealed one key potential site of dimeric interaction, with six residues <3Å apart. In particular, NIS residues Y242, T243, and Q471 were identified as critical to dimerization. Individual mutation of residues Y242 and T243 rendered NIS nonfunctional, while abrogation of Q471 did not impact radioiodide uptake. FRET data show that the putative dimerization interface can tolerate the loss of one, but not two, of these three clustered residues.

***Conclusions:*** We show for the first time that NIS dimerizes *in vitro*, and we identify the key residues via which this happens. We hypothesize that dimerization of NIS is critical to its trafficking to the PM and may therefore represent a new mechanism that would need to be considered in overcoming therapeutic failure in patients with thyroid cancer.

## Introduction

At least a quarter of patients with differentiated thyroid cancer (DTC) do not concentrate radioiodide (^131^I) sufficiently for effective ablation therapy (1,2), which remains a particular problem in metastatic disease. There are essentially two groups of thyroid cancer patients: those who respond to radioiodide treatment and therefore have an excellent prognosis, and those who do not respond and for whom outcome is dire (3). Troublingly, DTC is now the most rapidly increasing cancer in the United Kingdom and the United States, with ∼300,000 new cases reported worldwide per year. More than 40,000 people will die from thyroid cancer this year (4).

Mechanisms that influence treatment success in those thyroid cancers that are radioiodide resistant have been described in numerous investigations. While clinical progress is being made (5–8), the underlying causes of treatment failure are still not fully understood and the pathogenic processes associated with radioiodine resistance in individual patients or lesions need to be delineated.

Radioiodide uptake is mediated via the sodium/iodide symporter (NIS). Decreased levels of NIS expression and/or diminished targeting of NIS to the plasma membrane (PM) of thyroid cancer cells represent the principal mechanisms of radioiodide-refractory disease (9–11). Numerous studies have addressed the common pathways of NIS regulation *in vitro* and *in vivo* (12–17). Other studies have investigated the key transcriptional and epigenetic alterations that silence thyroid-specific genes such as *NIS* (15,18–21). To actively transport iodide for thyroid hormone biosynthesis and radioiodide treatment, NIS must be present in the basolateral PM of thyroid follicular cells. However, relatively little is known about the mechanisms that govern the trafficking of NIS or its intrinsic preference as a monomeric or multimeric protein.

Multiple membrane proteins are functionally regulated via dimerization (22–27), and circumstantial evidence has previously suggested that NIS may dimerize. For example, using freeze-fracture electron microscopy, intramembrane particles in NIS-expressing oocytes were deemed to be too large to be monomers (28).

The most detailed appraisal of the potential for NIS to dimerize was carried out by Huc-Brandt *et al.* (29). Electrophoresis patterns of NIS were suggestive of dimerization, and size exclusion chromatography and light scattering analyses also supported the notion that NIS may dimerize *in vitro* (29). In fact, the majority of NIS species had molecular weights corresponding to those of putative dimers and higher multimers, suggesting that NIS exists mainly in multimeric form (29). However, to what extent dimerization of NIS influences function and how this might impact upon radioiodide uptake in patients with thyroid cancer remain unclear.

We hypothesized that NIS dimerizes and that dimerization is critical to NIS function. We challenged the putative dimerization of NIS through three separate technologies and modeled potential sites of NIS:NIS interaction. Our data show that NIS does indeed dimerize and that abrogation of key dimeric residues renders NIS unable to transport iodide, findings that now warrant investigation in patients with DTC.

## Materials and Methods

### Cell lines

The SW1736 human anaplastic thyroid carcinoma cell line was kindly supplied by Dr. Rebecca Schweppe (University of Colorado) and maintained in RPMI 1640 medium (Thermo Fisher Scientific, Waltham, MA). The HeLa human cervical carcinoma cell line was acquired from European Collection of Authenticated Cell Cultures (ECACC, Porton Down, United Kingdom) and maintained in high-glucose Dulbecco's modified Eagle's medium (Sigma, St. Louis, MO). Both were supplemented with 10% fetal bovine serum (Thermo Fisher Scientific), penicillin (10^5^ U/L), and streptomycin (100 mg/L).

### Plasmids, transfection, and mutagenesis

The full-length human NIS cDNA was cloned in the pcDNA3.1+ vector with a C-terminal MYC (NIS-MYC) or HA (NIS-HA) tag (30). NIS-MYC and NIS-HA were both required for the coimmunoprecipitation (co-IP) and proximity ligation assays (PLAs), which necessitated two distinct tags. For use in the Förster resonance energy transfer (FRET) experiments, NIS cDNA was inserted into the *Hin*dIII and *Bam*HI restriction sites of the cerulean-N1 and citrine-N1 vectors (gifts from Michael Davidson & Dave Piston obtained from Addgene, Watertown, MA) to create *NIS* constructs conjugated at the C-terminus to a cerulean or citrine fluorophore, respectively.

Transfections were performed with TransIT^®^-LT1 reagent (Geneflow, Lichfield, United Kingdom) following the manufacturer's protocol at a 3:1 reagent to DNA ratio and experiments performed after 48 hours. Specific mutations were made as indicated using the QuikChange II XL Site-Directed Mutagenesis Kit (Agilent Technologies, Santa Clara, CA).

### Immunofluorescence staining and PLA

Immunofluorescence staining was conducted as described previously (30). Primary antibodies used were mouse monoclonal anti-MYC-Tag 9B11 (1:750; Cell Signaling Technology, Danvers, MA), rabbit monoclonal anti-HA Y-11 (1:100; Santa Cruz, Dallas, TX), mouse monoclonal anti-HA 16B12 (1:100; BioLegend, San Diego, CA), rabbit monoclonal anti-Na^+^/K^+^/ATPase (Alexa Fluor^®^ 488), EP1845Y (1:50; Abcam, Cambridge, United Kingdom), and rabbit monoclonal anti-Na^+^/K^+^/ATPase EP1845Y (1:250; Abcam).

A Zeiss LSM 510 confocal microscope with × 40 objective was used to perform confocal microscopy (Carl Zeiss AG, Oberkochen, Germany). Epifluorescent microscopy was performed using × 40 objective on a Leica DM6000 fluorescent microscope (Leica Microsystems, Wetzlar, Germany). The Duolink *In Situ* Fluorescence Protocol with Detection Reagents Red Kit (Sigma) was used as per the manufacturer's instructions. Cells were transfected for 48 hours before fixation, permeabilization, and addition of anti-MYC and anti-HA antibodies.

### Western blotting

Cells were harvested in RIPA (50 mM Tris-HCl, pH 7.4, 150 mM NaCl, 1% vol/vol Igepal CA-630, 6 mM sodium deoxycholate, 1 mM EDTA) with a protease inhibitor cocktail (Sigma). Western blotting was performed as described previously (31). Proteins (20 μg) were separated by SDS-PAGE (sodium dodecyl sulfate–polyacrylamide gel electrophoresis) using 12% acrylamide gels. Primary antibodies used were rabbit polyclonal anti-NIS (1:1000; Proteintech, Rosemont, IL), mouse monoclonal anti-MYC-Tag 9B11 (1:1000; Cell Signaling Technology), rabbit monoclonal anti-HA Y-11 (1:1000; Santa Cruz), and mouse monoclonal β-actin AC-15 (1:10,000; Sigma).

### Radioiodide uptake assays

Cells were seeded into 24-well plates and transfected as above. Radioiodide uptake assays were performed 48 hours after transfection as described previously (32). Briefly, 1 μM NaI and 0.05 μCi ^125^I were added directly to the cell medium. After incubation at 37°C for 1 hour, medium was removed and cells were washed rapidly with Hanks' balanced salt solution. Cells were lysed in 2% SDS, and the radioactivity of the lysate was counted for one minute in a gamma counter. Results are given as picomoles of I^−^ per microgram of protein. To demonstrate specific uptake, the NIS inhibitor sodium perchlorate was used at 100 μM to pretreat control wells for 1 hour before the addition of radioiodide.

### Homology modeling of NIS dimerization

The 3D structure of the NIS sequence was generated in Phyre2, based on two protein templates: the two dimeric vSGLT structures [PDB ID: 2XQ2 (33) and 3DH4 (34)] were used with 100% confidence of sequence homology between NIS and template sequence. Using these templates, 74% NIS residues were modeled at >90% confidence, with 95 residues modeled *ab initio*. Subsequent alignment of this NIS structure with the crystal structure of vSGLT [PBD ID: 2XQ2 (33)] in YASARA revealed a homology model, with a root-mean-square deviation (RMSD) of atomic positions of 1.178Å, indicating a relatively small distance between the backbone atoms of the two aligned protein structures. To improve the homology model of NIS dimerization, the regions of NIS sequence that could not be aligned onto the vSGLT crystal structure needed to be identified and removed.

To achieve this, six different topology predictions were performed on the NIS primary sequence to identify which regions of NIS are likely to form transmembrane domains (TMDs), as TMDs are well structured and therefore more accurate to model. To improve homology between the two proteins, the large intracellular C-terminal tail of NIS (from S556 onward) was removed. To further improve homology, the large extracellular domain between TMDs 12 and 13 (from T472 to Y524) was also removed from the NIS sequence. The 3D structure of this modified NIS sequence, generated as above, gave a more refined homology model of NIS structure, with 93% residues modeled at >90% confidence and just 33 residues modeled *ab initio*, and an RMSD of 0.993Å.

### Förster resonance energy transfer

Cells were seeded in eight-well chambered cover glass (Lab-Tek Nunc; Thermo Fisher Scientific) and cotransfected with NIS variants conjugated to either cerulean or citrine. FRET imaging was performed 48 hours post-transfection using Crest X-Light spinning disk head coupled to a Nikon Ti-E automated base and 60 × /1.40 NA objective. Confocal microscopy was performed and three concurrent versions of each image were captured: total cerulean image (cerulean excitation/cerulean emission [CFP/CFP]), total citrine image (citrine excitation/citrine emission [YFP/YFP]), and FRET image (cerulean excitation/citrine emission [CFP/YFP]). Excitation was delivered at λ = 430–450 nm for cerulean and at λ = 500–520 nm for citrine using a Lumencor Spectra X Light Engine, and emitted signals detected at λ = 460–500 nm for cerulean and at λ = 535–565 nm for citrine using a Photometrics Evolve Delta 512 EMCCD.

Data analysis was performed using MetaMorph^®^ Version 7.8.13.0 software (Molecular Devices, Wokingham, United Kingdom). Background subtraction was performed on images before further analysis. Controls were carried out to determine YFP and CFP cross talk into the FRET image. Cells expressing citrine alone were imaged as above. A region of interest (ROI) was drawn around each cell and the ratio of the average gray level of the ROI in the FRET image to that in the YFP image was calculated as per Equation (a) below to determine YFP cross talk. To determine CFP cross talk, cells expressing cerulean alone were imaged as above and the ratio of the average gray level of the ROI in the FRET image to that in the CFP image was calculated as per Equation (b). (a) YFP cross talk = FRET image ÷ YFP image; (b) CFP cross talk = FRET image ÷ CFP image. Once the extent of YFP and CFP cross talk into the FRET image was established, Equation (c) below was then used to correct for YFP and CFP cross talk in cells expressing both fluorophores. (c) Corrected FRET = FRET image − (YFP image × YFP cross talk) − (CFP image × CFP cross talk).

### Statistical analyses

Data were analyzed using one-way ANOVA with Dunnett's multiple comparison test to compare with wild type (WT), or Tukey's multiple comparison test to compare all means. If data were nonparametric, Kruskal–Wallis multiple comparison test was used instead. Two-way ANOVA with Sidak's multiple comparison test was also used. Significance was taken as *p* < 0.05.

## Results

### co-IP of dimeric NIS *in vitro*

**FIG. 1.**
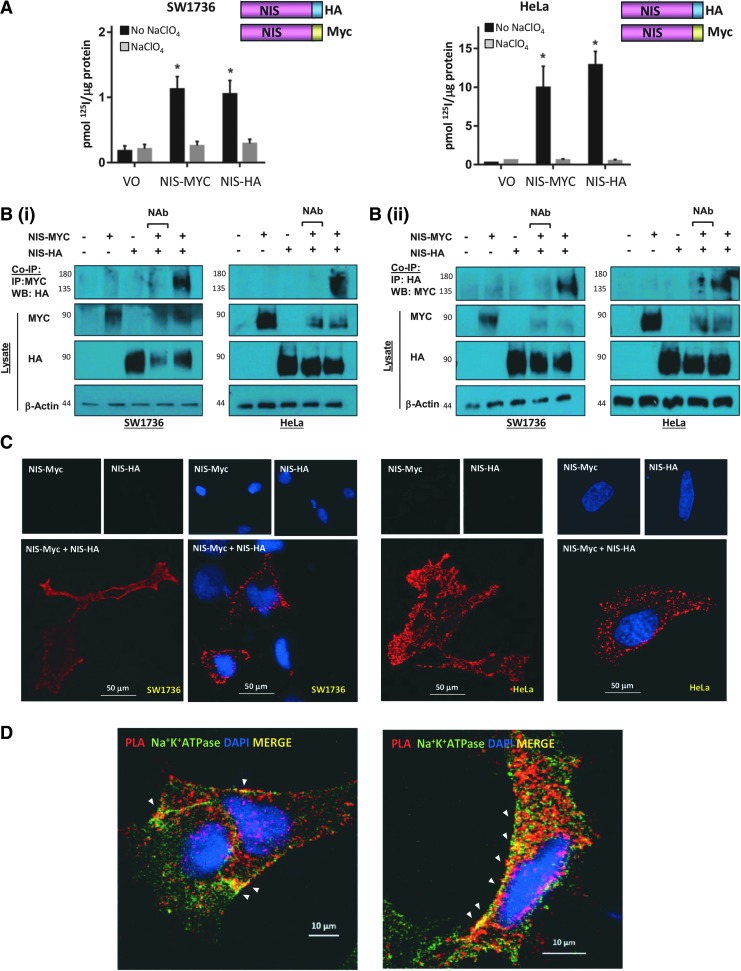
**(A)** Cells were transfected with empty pcDNA3.1+ vector (VO) or differentially tagged NIS variants and radioiodide uptake assays performed. Significant radioiodide uptake compared with VO was observed with both MYC- and HA-tagged NIS variants. Pretreatment with sodium perchlorate (NaClO_4_) to inhibit NIS (gray bars) demonstrates NIS-specific iodide uptake in black bars. Bars show mean radioiodide uptake in pmol ^125^I/μg protein ± SEM. **(B)** Representative co-IP assays demonstrating direct interaction between NIS-MYC and NIS-HA in SW1736 and HeLa cells. Cells were transfected with VO (lane 1), VO+NIS-MYC (lane 2), VO+NIS-HA (lane 3), or NIS-MYC+NIS-HA (lanes 4 and 5) and lysed. Co-IP was performed on all lysates (except no antibody control, lane 4) with mouse anti-MYC **(i)** or rabbit anti-HA **(ii)**. Western blot using antibody to the other tag detected the presence of the other NIS variant, demonstrating a direct interaction between the two NIS variants (co-IP blots, top panel). **(C)** PLAs with and without DAPI staining for nuclei (blue) demonstrating close proximity (<40 nm) between NIS-MYC and NIS-HA throughout the cell, including the PM. **(D)** PLAs with Na^+^K^+^ATPase immunofluorescent staining. Areas of colocalization (yellow; white arrowheads) between PLA signal generated by close proximity of NIS-MYC and NIS-HA (in red) and the PM marker Na^+^K^+^ATPase (in green) in representative SW1736 (left) and HeLa (right) cells were apparent. *p < 0.05. co-IP, coimmunoprecipitation; NIS, sodium/iodide symporter; PLAs, proximity ligation assays; PM, plasma membrane; SEM, standard error of the mean.

We used three separate strategies to evaluate the potential NIS:NIS interaction, (i) co-IP assays, (ii) PLAs, and (iii) FRET assays, and utilized both a thyroid (SW1736) and nonthyroid (HeLa) cell line. First, we tagged NIS with either an HA or an MYC tag on the N- or C-terminus. Initial experiments showed that C-terminal tagging was generally preferable to N-terminal tagging in terms of protein detection, normal subcellular localization, and impact upon radioiodide uptake (data not shown). NIS tagged C-terminally with HA or MYC was functional in SW1736 and HeLa cells (Fig. 1A), and yielded canonical unglycosylated (∼60 kDa) and glycosylated (∼75 to ∼90 kDa) monomeric forms under reducing conditions of Western blotting (Supplementary Fig. S1).

We then progressed to co-IP assays in SW1736 and HeLa cells. When we pulled back with an anti-MYC antibody and immunoblotted using an anti-HA antibody, we observed a band of between 135 and 180 kDa in lanes with NIS-MYC and NIS-HA coexpression, but not in control lanes (Fig. 1B-i). A band of a similar size was often observed in the lysate of cells transfected with either NIS-MYC or NIS-HA through Western blotting and is therefore likely to demonstrate the presence of NIS-MYC:NIS-MYC and NIS-HA:NIS-HA homodimers (Supplementary Fig. 2).

Reciprocal co-IPs, where we immunoprecipitated with anti-HA antibody and probed using anti-MYC antibody, revealed consistent findings with dimeric bands of ∼135 to 180 kDa in lanes with NIS-MYC and NIS-HA coexpression, but not in control lanes (Fig. 1B-ii). Thus, forward and reverse co-IP assays in two cell lines reveal the apparent interaction between NIS-MYC and NIS-HA heterodimers.

### PLA determination of the subcellular sites of NIS dimerization

Next, we challenged our findings through PLAs, which detect subcellular proximity between proteins if they are separated by fewer than 40 nm (35,36). Using anti-MYC and anti-HA antibodies in SW1736 and HeLa cells transfected with both NIS-MYC and NIS-HA, red spots of specific NIS:NIS interaction were apparent, whereas no binding was detected in cells transfected with NIS-MYC or NIS-HA alone (Fig. 1C). Confocal microscopy revealed the subcellular localization of NIS-HA:NIS-MYC interaction to be predominantly cytoplasmic, with some clear PM binding.

To address PM localization more specifically, we performed PLAs and then carried out confocal immunofluorescent microscopy in the same fixed cells (Fig. 1D). Staining for the sodium/potassium adenosine triphosphatase (Na^+^/K^+^-ATPase), a solute pump that regulates intracellular sodium and potassium at the PM and hence is critical to NIS function, revealed discrete areas of colocalization at the PM, where the Na^+^/K^+^-ATPase is predominantly active. However, colocalization between the dimeric NIS (NIS-MYC:NIS-HA) and Na^+^/K^+^-ATPase was also apparent in intracellular compartments, suggesting that NIS does not exist as a dimer exclusively at the PM.

### Quantification of NIS dimerization via FRET

**FIG. 2.**
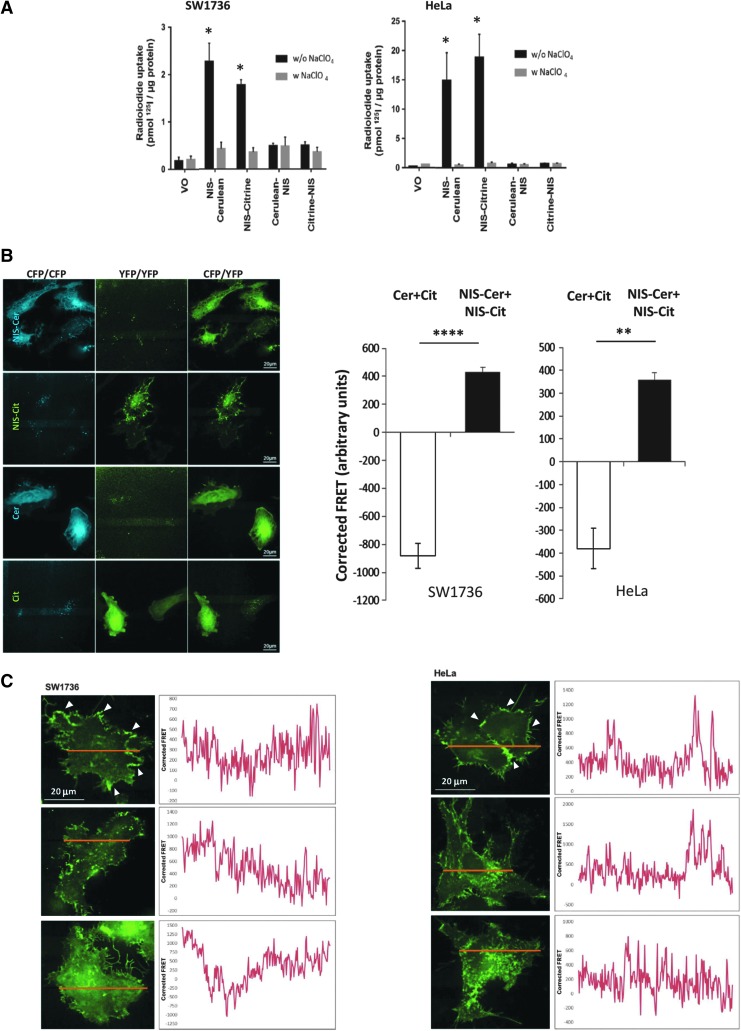
**(A)** SW1736 (left) and HeLa (right) cells were transfected with VO or one differentially tagged NIS variant and radioiodide uptake assays were performed. Pretreatment with sodium perchlorate (w. NaClO_4_) was used to specifically inhibit NIS activity (gray bars). **(B)** Representative images from FRET analysis of SW1736 cells transfected with cerulean+citrine (Cer+Cit, negative control) and NIS-cerulean+NIS-citrine (NIS-Cer+NIS-Cit; left). Right*—*coexpression of both NIS-fluorophore constructs gave significantly greater corrected FRET signals than the negative control, demonstrating a close proximity between the two fluorophores that is indicative of dimerization. Bars show mean corrected FRET signal in arbitrary units and error bars show SEM. One-way ANOVA with Tukey's multiple comparison statistical tests was performed: **p < 0.01, ****p < 0.001 (*n* = 5 with 10 individual cells in each *n*). **(C)** SW1736 (left) and HeLa (right) cells were transfected with NIS-cerulean+NIS-citrine and FRET analysis was performed on linear sections across the cell (image). Arrowheads indicate examples of prominent regions of NIS dimerization at the PM. Graphs depict the corrected FRET signal across the lines, suggesting that NIS dimerization occurs throughout the cell (*n* = 5 cells measured per experiment). *p < 0.05. FRET, Förster resonance energy transfer.

Having shown through co-IP assays and PLAs that we could detect the specific binding of HA-tagged and MYC-tagged NIS *in vitro*, we next carried out FRET assays as a final confirmatory method of detecting NIS dimerization. Furthermore, through using confocal microscopy to detect the transfer of fluorescent light energy between bound proteins that are within 10 nm of each other, FRET assays would facilitate the quantification of NIS dimerization. We made constructs conjugating the fluorophore citrine (Cit) or cerulean (Cer) to the C- and N-termini of NIS and assessed NIS functionality. Only C-terminally tagged NIS elicited significant radioiodide uptake (Fig. 2A) and yielded the fully processed NIS protein (Supplementary Fig. S3).

After correcting for cross talk between the citrine and cerulean fluorophores, which was calculated as 23.1% and 43.7%, respectively (see the Materials and Methods section), coexpression of the NIS-fluorophore constructs (*NIS-Cer* + *NIS-Cit*) resulted in a significant increase in corrected FRET signal (arbitrary units) compared with negative control cells (*CerCit*) expressing both the free fluorophores (SW1736: 430.62 ± 32.74 [NIS-CerCit] vs. −882.00 ± 88.16 [CerCit], *p* < 0.001; HeLa: 357.60 ± 33.50 [NIS-CerCit] vs. −380.54 ± 134.00 [CerCit], *p* < 0.01; Fig. 2B). Thus, fluorophores were only able to undergo FRET when they were conjugated to NIS, signifying that the two NIS monomers were in close enough proximity to dimerize. Two positive control systems for FRET confirmed specific detection of binding (Supplementary Fig. S4). FRET data hence confirmed our co-IP and PLA findings of NIS dimerization.

To elucidate the subcellular location of NIS dimerization, FRET analysis was performed on linear sections across SW1736 and HeLa cells cotransfected with NIS-cerulean and NIS-citrine and plotted against distance along the line. Although there were clear regions of intense apparent dimerization at the PM (Fig. 2C; arrowed in top panel), on average the FRET signal appeared to be distributed fairly evenly across the width of *N* = 20 cells assessed. FRET data were therefore consistent with PLA findings in that NIS dimerization occurs both in the cytosol and at the PM.

### Predicting the key amino acid residues for NIS dimerization

As co-IP, PLA, and FRET data had all supported the notion that NIS does in fact dimerize, we set about trying to identify the specific regions or motifs of NIS responsible for interaction. While not empirically investigated previously, a leucine zipper motif is apparent in TMD 6 of NIS (37) and a putative glycine zipper motif (GZM) in TMD 12. However, while abrogation of each domain resulted in a decreased ability to uptake radioiodide in both SW1736 and HeLa cells, PLA and FRET demonstrated that leucine and glycine zipper mutants retained the ability to dimerize (Supplementary Fig. S5).

**FIG. 3.**
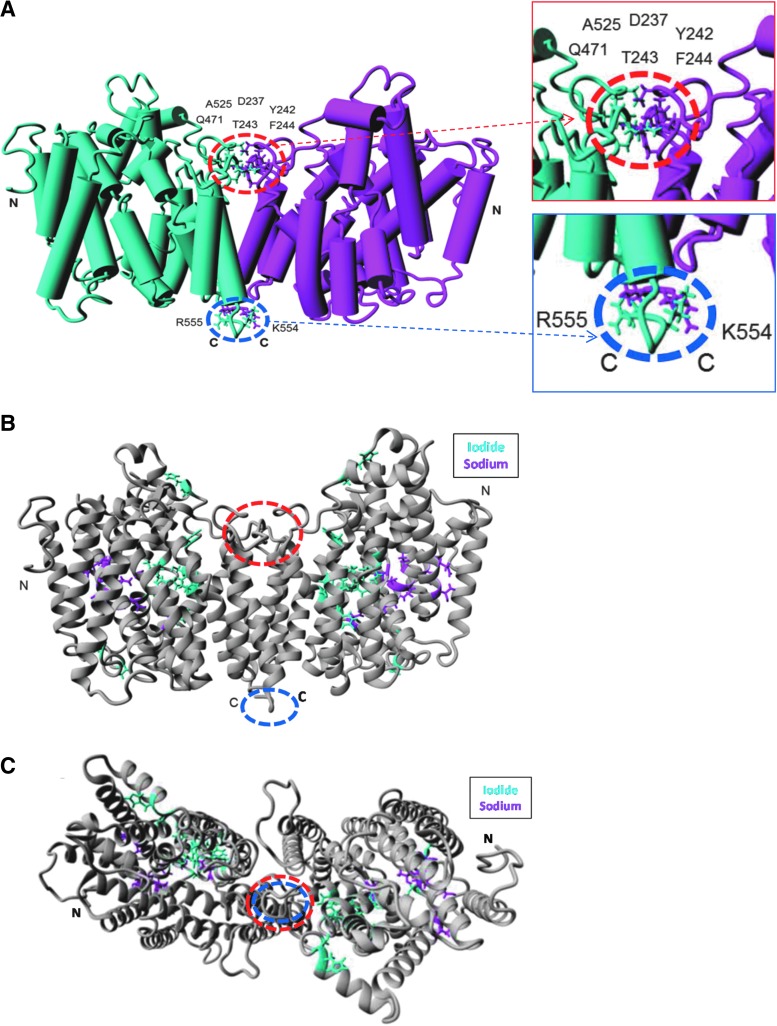
**(A)** Eight novel residues were identified as potentially involved in NIS dimerization using the refined NIS homology model, appearing to form two distinct putative dimerization interfaces, a larger (“primary”) region circled in red and smaller (“secondary”) region in blue. Side-chains of residues found to be <3Å from the opposing monomer are shown in “Stick” form, and monomers are colored cyan or magenta. Structures are viewed in the membrane plane oriented so the extracellular milieu is above the structure. Amino and carboxy termini are labeled (N and C, respectively). Inset*—*magnified regions of closest interaction. **(B)** Residues known to be involved in sodium or iodide binding and are shown in “Stick” form and colored in magenta and cyan, respectively, while remaining residues are colored in gray. Structures are viewed in the membrane plane oriented so the extracellular milieu is above the dimer. **(C)** The putative dimeric structure viewed from the extracellular side.

To therefore identify *de novo* residues involved in dimerization, a homology model of NIS structure based on the crystal structure of the bacterial protein vSGLT was built using the modeling platform Phyre2 (see the Materials and Methods section for details). The crystal structure of the solute transporter vSGLT (34) has been used previously to model monomeric NIS (38). As the crystal structure of vSGLT is dimeric, we then threaded NIS residues on to this to create a 3D model of dimerized NIS. Our modeling identified eight residues predicted to be fewer than 0.3 nm from the opposing monomer (D237, Y242, T243, F244, Q471, A525, K554, R555; Fig. 3A). Interestingly, these amino acids were not directly proximal to residues previously shown to be critical to iodide or sodium transport (Fig. 3B, C). This therefore hints that dimerization of NIS is not required as a structural prerequisite for iodide uptake.

### The functional consequences of abrogating putative residues implicated in dimerization

**FIG. 4.**
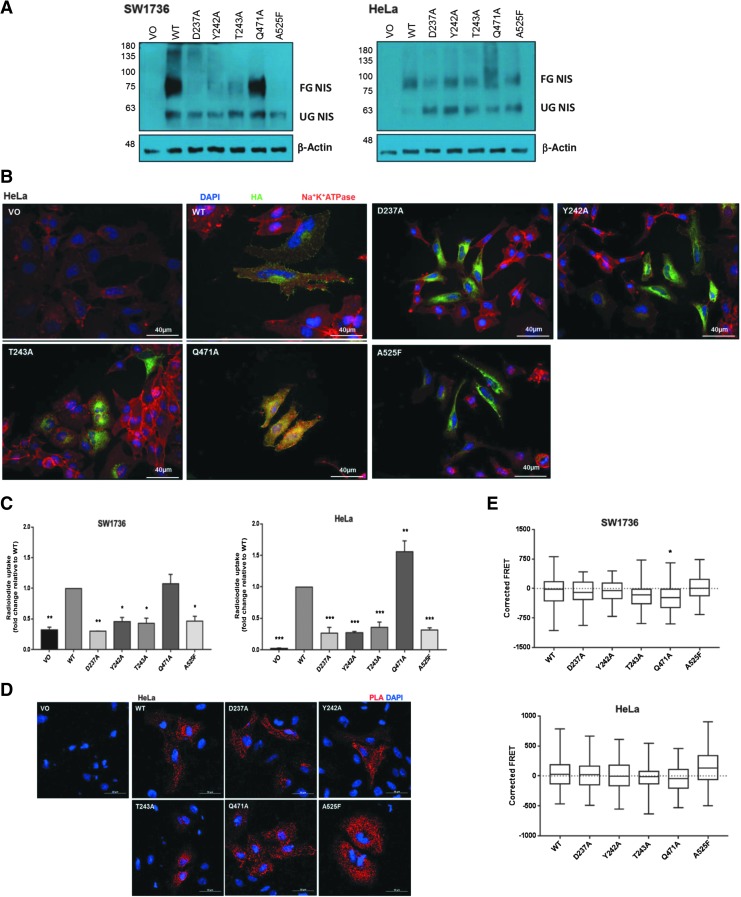
**(A)** Mutating residues within the putative dimerization interface alter NIS glycosylation. Western blots were performed on SW1736 and HeLa cell lysates with HA-tagged WT and mutant NIS expression using the mouse anti-NIS antibody. A band at 75–100 kDa represents the fully glycosylated mature form of NIS, while a band at ∼60 kDa represents the nonglycosylated immature form of NIS. **(B)** NIS localization of HA-tagged NIS mutation of individual residues of the putative dimerization interface. Immunofluorescence analysis was performed using the mouse anti-HA primary antibody to visualize NIS (green), while the rabbit anti-Na^+^/K^+^/ATPase primary antibody was used as a PM marker (red). Nuclei are visualized in blue using Hoechst stain, 40 × magnification. **(C)** Radioiodide uptake is abolished when residues within the putative dimerization interface are mutated, with the exception of Q471A, in SW1736 and HeLa cells. Bars represent mean relative radioiodide uptake relative to WT ± SEM. *p < 0.005, **p < 0.0005, and ***p < 0.0001 compared with WT (*n* = 3 with 4 replicates in each *n*). **(D)** PLA signal is still observed in HeLa cells when single residues in the putative dimerization interface are mutated, 40 × magnification. **(E)** Corrected FRET values when residues of the putative dimerization interface were mutated. Data presented as box and whisker plots, with the center line indicating the mean corrected FRET value (in arbitrary units), the box representing the 25th and 75th percentiles, and the whiskers denoting the minimum and maximum values. *p < 0.05. *n* = 2, with a minimum of 20 cells analyzed in each *n*. WT, wild type.

Based on the modeling of Figure 3, we considered the region containing D237, Y242, T243, F244, Q471, and A525 to be a “hot-spot” of potential dimerization, and to identify whether these residues might be involved in dimerization, we made the following point mutations: D237A, Y242A, T243A, Q471A, and A525F. With the exception of Q471A, which had a similar banding pattern to WT, the proportion of glycosylated versus nonglycosylated NIS was visibly lower for all mutants compared with WT, suggesting that mutating the majority of the putative dimerization interface residues impairs NIS protein maturation (Fig. 4A and Supplementary Fig. S6), although expression and glycosylation patterns were variable between the two cell lines under our standard reducing Western blotting conditions.

Immunofluorescence microscopy using anti-Na^+^/K^+^/ATPase as a PM marker revealed that while WT and Q471 showed PM localization, no membrane localization was observed for the mutants D237A or A525F (Fig. 4B and Supplementary Fig. S7). Similarly, the vast majority of Y242A and T243A NIS protein was intracellularly retained. Collectively these data imply that mutations in the putative dimerization interface usually impair the maturation of NIS and its trafficking to the PM, with the exception of Q471A.

Functionally, the radioiodide uptake of mutant Q471A was similar to or greater than WT in both cell lines (Fig. 4C), whereas uptake of the remaining mutants was significantly lower than WT. This appears to reflect the expression of the fully glycosylated, mature form of NIS (Fig. 4A and Supplementary Fig. S6). Finally, NIS dimerization was assessed via PLA and FRET in SW1736 and HeLa cells. All mutants retained the ability to homodimerize (Fig. 4D), although Q471A showed a marginal decrease in whole-cell FRET signal in SW1736s (Fig. 4E). Thus, mutation of individual residues, which lie within this putative region, does not impede the ability of NIS monomers to homodimerize but may impair NIS maturation and, consequently, function.

### The putative dimerization interface can tolerate the loss of one, but not two, of the three clustered residues

**FIG. 5.**
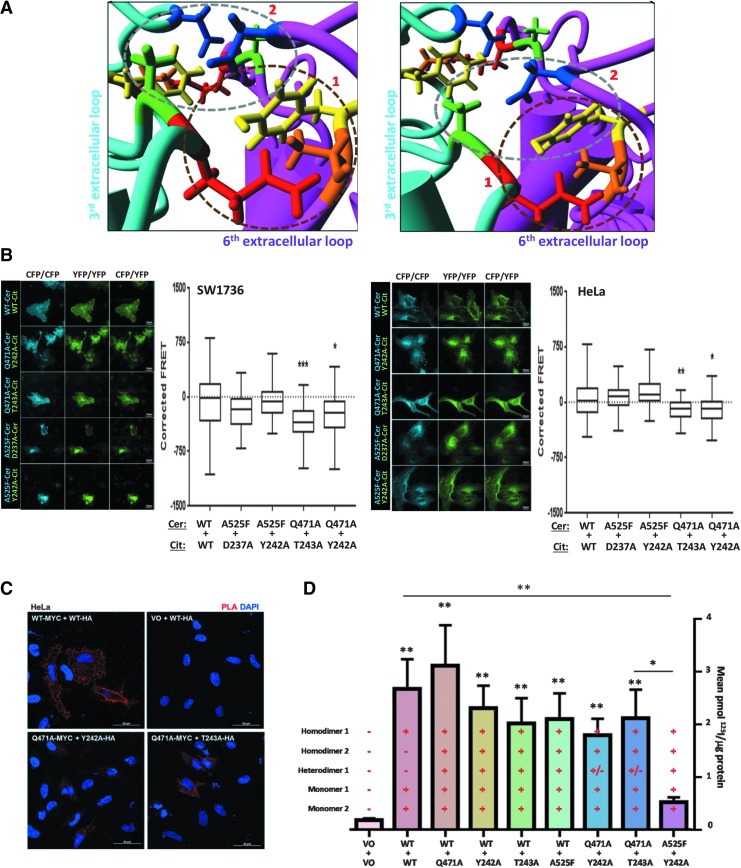
**(A)** Detailed examination of the primary putative dimerization interface. Two distinct clusters of residues are particularly close and are shown in different planes of view. Cluster 1 (circled in brown) comprises Q471 (red) of the cyan monomer and Y242 (yellow) and T243 (orange) of the opposite magenta monomer. Cluster 2 (circled in gray) comprises A525 (green) of the cyan monomer and D237 (blue) and potentially Y242 of the opposite magenta monomer. **(B)** FRET analysis of combined putative dimerization interface mutants in SW1736 and HeLa cells cotransfected with differentially tagged (either cerulean or citrine) variants of WT NIS or a combination of two NIS mutants (*n* = 2, with a minimum of 20 cells analyzed in each *n*). Representative images captured during FRET analysis are shown. Three concurrent versions of each image were taken: total cerulean (CFP) image (cerulean excitation/cerulean emission [CFP/CFP]), total citrine (YFP) image (YFP excitation/YFP emission [YFP/YFP]), and the FRET image (cerulean excitation/citrine emission [CFP/YFP]). **(C)** Reduced PLA signal was observed with combinations of mutations within the NIS putative dimerization interface in HeLa cells, 40 × magnification. (**D**) Radioiodine uptake in SW1736 cells in response to various combinations of cotransfected WT and mutant NIS. As indicated, each bar represents the possibility of multiple simultaneous monomeric and dimeric NIS species. **p < 0.01, compared with WT:WT. *N* = 3 experiments. *p < 0.05, **p < 0.01, ***p < 0.001.

“Stick” modeling of amino acid residues revealed that the putative dimerization interface comprises residues on two separate extracellular loops: the third (D237, Y242, and T243) and the sixth (Q471 and A525) loops. Residues on the third extracellular loop on one monomer (in magenta) are in closest proximity to those of the sixth extracellular loop on the opposite monomer (in cyan; Fig. 5A). Of these residues, Q471 (red), Y242 (yellow), and T243 (orange) seem to cluster together ([1]; circled in brown; Fig. 5A), while A525 (green) and D237 (blue) appear to form a separate cluster ([2]; circled in gray).

Acting upon this apparent proximity, FRET was carried out on combinations of residues that formed the most proximal pairings. A statistically significant reduction in corrected FRET compared with WT was observed for two of the combinations in both cell lines: Q471A+Y242A (SW1736: mean − 241.68, range −997.58 to 411.97, *p* < 0.05; HeLa: mean − 82.14, range −516.51 to 354.33, *p* < 0.05) and Q471A+T242A (SW1736: mean − 349.69, range −986.66 to 168.42, *p* < 0.001; HeLa: mean − 104.45, range −424.75 to 158.84, *p* < 0.01; Fig. 5B). However, there was no significant change in corrected FRET compared with WT for the remaining two combinations of A525F+D237A or A525F+Y242A in either cell line. Representative FRET images are given in Figure 5B.

While not quantified empirically, PLA demonstrated similarly reduced red spots of dimerization for the combinations of Q471A+Y242A and Q471A+T242A compared with WT (Fig. 5C). Collectively, these observations suggest that the interactions between residues Q471A, Y242A, and T243A within the primary putative dimerization interface are critical for NIS dimerization, and mutation of the most proximal residues of extracellular loops 3 and 6 significantly impairs dimerization.

### NIS function in response to disrupted dimerization

Finally, we assessed the functional impact of altering the ability of NIS to dimerize, although these experiments are complicated by the fact that up to five different NIS species theoretically exist in each individual combination treatment (one homodimer of each NIS mutant; one heterodimer; and one monomer of each mutant). We first examined each individual mutation (Q471A, Y242A, T243A, and A525F) in combination with WT. Our data of Figure 4D show that Q471A retains radioiodide uptake, while Y242A, T243A, and A525F all show significantly impaired function. When combined with WT NIS, Q471A gave radioiodide uptake values indistinguishable from WT in SW1736 cells (Fig. 5D), whereas Y242A, T243A, and A525F showed nonsignificantly reduced uptake. Consistent data were apparent in HeLa cells (Supplementary Fig. S8).

When we appraised the functional consequence of NIS mutants that FRET had revealed to show significantly reduced heterodimerization, we discovered that there was no significant impact on radioiodide uptake (Fig. 5D) unless two nonfunctional mutants were combined (A525F+Y242A), where iodide uptake was markedly lost (Fig. 5D).

We thus propose the following potential rules: (i) NIS dimerization is relatively “strong” and needs at least two matching and opposing mutations to disrupt it. (ii) While NIS does not need to be fully processed or glycosylated to dimerize, this is the predominant dimeric form under WT conditions, based on co-IP data; (iii) For appreciable ^125^I uptake, at least one NIS monomer must be fully processed/glycosylated (e.g., Q471A+Y242A); if neither are fully processed, then no radioiodide uptake is possible, despite the fact that the monomers can dimerize (e.g., A525F+Y242A). (iv) Dimerization may potentially occur before processing/glycosylation. Subsequently, if at least one of the monomeric NIS molecules is processed/glycosylated, the dimer can then be trafficked to the PM where radioiodide uptake can occur.

## Discussion

The factors regulating the function of NIS are of direct clinical importance due to its central role in radioiodide treatment; patients with radioiodide-refractory (RAIR) thyroid cancer, particularly those with metastatic disease, have a life expectancy of 3–5 years and represent a group for whom there remains a clear unmet medical need (1,2). TSH (thyrotropin) induces iodide uptake through upregulation of NIS expression and the modulation of its subcellular localization (39–41). Many thyroid cancers demonstrate reduced NIS activity through diminished PM retention (42–44). BRAF-mutant tumors (60–70% of thyroid cancers) are more likely to be resistant to radioiodide, partly due to decreased NIS expression (13,45), but also due to impaired PM targeting (11,14) through mechanisms that remain ill-defined. Indeed, PTTG1 Binding Factor (PBF;PTTG1IP) is the only protein currently known to bind to NIS and modulate its subcellular localization (30).

Numerous PM proteins have been reported to dimerize and for this to be integral to their trafficking and/or PM function (22–27,29). However, although several previous studies have directly or indirectly addressed the putative dimerization of NIS, the evidence to date remains circumstantial, particularly as they have relied on the use of extracted proteins and cannot exclude the possibility of nonspecific protein aggregation. We therefore sought to determine whether NIS does in fact dimerize, what structure it forms, and what its impact might be on its canonical function.

For the first time, we show via three discrete methodologies that NIS does in fact dimerize *in vitro*. Co-IP, PLA, and FRET data all consistently revealed abundant and detectable cellular dimerization of NIS. This was not confined to particular cellular compartments, but appeared to occur generally within the cytoplasm, as well as at the PM. It is not clear from these studies whether NIS dimerizes early in its trafficking to the PM, or whether it internalizes from the PM as a dimer.

Indeed, when we used FRET to quantify the intensity of dimerized NIS in cross sections of cells, no clear patterns emerged in keeping with PLA data. In our experience, NIS does not appear uniformly at the PM, but rather in discrete regions of higher intensity (30,32). Rapid spiking of peaks and troughs in the FRET signal may reflect vesicles of NIS being trafficked through the cell, possibly suggesting that NIS may be trafficking as a dimer. Therefore, our hypothesis is that NIS dimerizes in the endoplasmic reticulum (ER), progresses to the Golgi where various posttranslational modifications occur, and then is preferentially trafficked as a dimer to the PM.

Another member of the SLC5A protein family, the high-affinity choline transporter (CHT1), has been demonstrated to dimerize. The authors proposed that this dimerization may be mediated by a GZM within TMD 12 as this motif is highly conserved in members of the SLC5A family (46), including NIS where the putative GZM comprised glycine residues at positions 444 and 448. However, our data reveal that mutating the putative leucine and GZMs did not alter NIS dimerization and our homology model of NIS dimerization revealed that they were not in close proximity to our proposed sites of monomer:monomer binding. As these dimerization motifs are known to be important for helical packing (47), and as our mutants of these motifs resulted in proteins that could no longer be processed and trafficked to the PM, it is possible that these motifs are instead crucial for the proper folding of the TMDs of the NIS monomer.

In assessing the function of NIS mutants that cannot dimerize, radioiodide uptake findings are hampered by the obvious difficultly in discerning the individual physiological effects of monomeric and dimeric NIS. In combining mutants of the dimerization interface (e.g., Q471A+T243A, which gave the lowest FRET signal), individual mutants are still able to homodimerize (i.e., Q471A:Q471A; T243A:T243A) and will also exist as monomers (Q471A, T243A). Furthermore, it is difficult to know whether a nonfunctional mutant (e.g., A525F), which does not reach the PM, loses function because of a lack of dimerization or else nonspecifically due to altered cellular processing (such as ER-associated degradation, nonsense-mediated decay, and protein instability).

Thus, experiments designed to determine whether the significantly impaired dimerization of these two mutants specifically alters iodide transport are encumbered by multiple other protein events occurring simultaneously in the cell. Until we can specifically define the effect of NIS dimerization on its function, its importance remains to be fully determined.

However, what our data do show is that NIS dimerization is contingent upon three key residues: Y242 and T243 of one monomer, and Q471 of the other. A single mutation of one of these residues is insufficient to prevent interaction, but a combined mutation of Y242+Q471 or T243+Q471 results in significantly impaired interaction. Q471A retained iodide uptake activity and was correctly targeted to the PM, suggesting that individual mutations within the putative dimerization interface do not necessarily preclude PM targeting. Indeed, while the Y242A and T243A mutants were largely intracellularly retained, a small proportion of both mutants were still apparent via immunofluorescence microscopy at the PM.

In summary, we show that NIS can exist as a monomeric and dimeric protein *in vitro*, substantiating earlier circumstantial evidence (28,29,42,48,49). We propose that residues on the third extracellular loop of one NIS monomer are in closest proximity to those of the sixth extracellular loop on the opposite monomer and that this is where binding occurs. The role of dimerization remains unclear, but in keeping with other PM proteins (22–27), we hypothesize that dimerization aids trafficking to the PM. Whether patients with RAIR thyroid cancer show impaired dimerization of NIS and whether this contributes to the frequently observed reduction in NIS localization to the PM in DTC now need to be determined.

## Supplementary Material

Supplemental data

Supplemental data

Supplemental data

Supplemental data

Supplemental data

Supplemental data

Supplemental data

Supplemental data
