# Supplementary figures and images for: Dimerization of the Sodium/Iodide Symporter
Source: Thyroid. 2019 Oct 15;29(10):1485–98. doi: 10.1089/thy.2019.0034 (PMC6797079; doi:10.1089/thy.2019.0034)

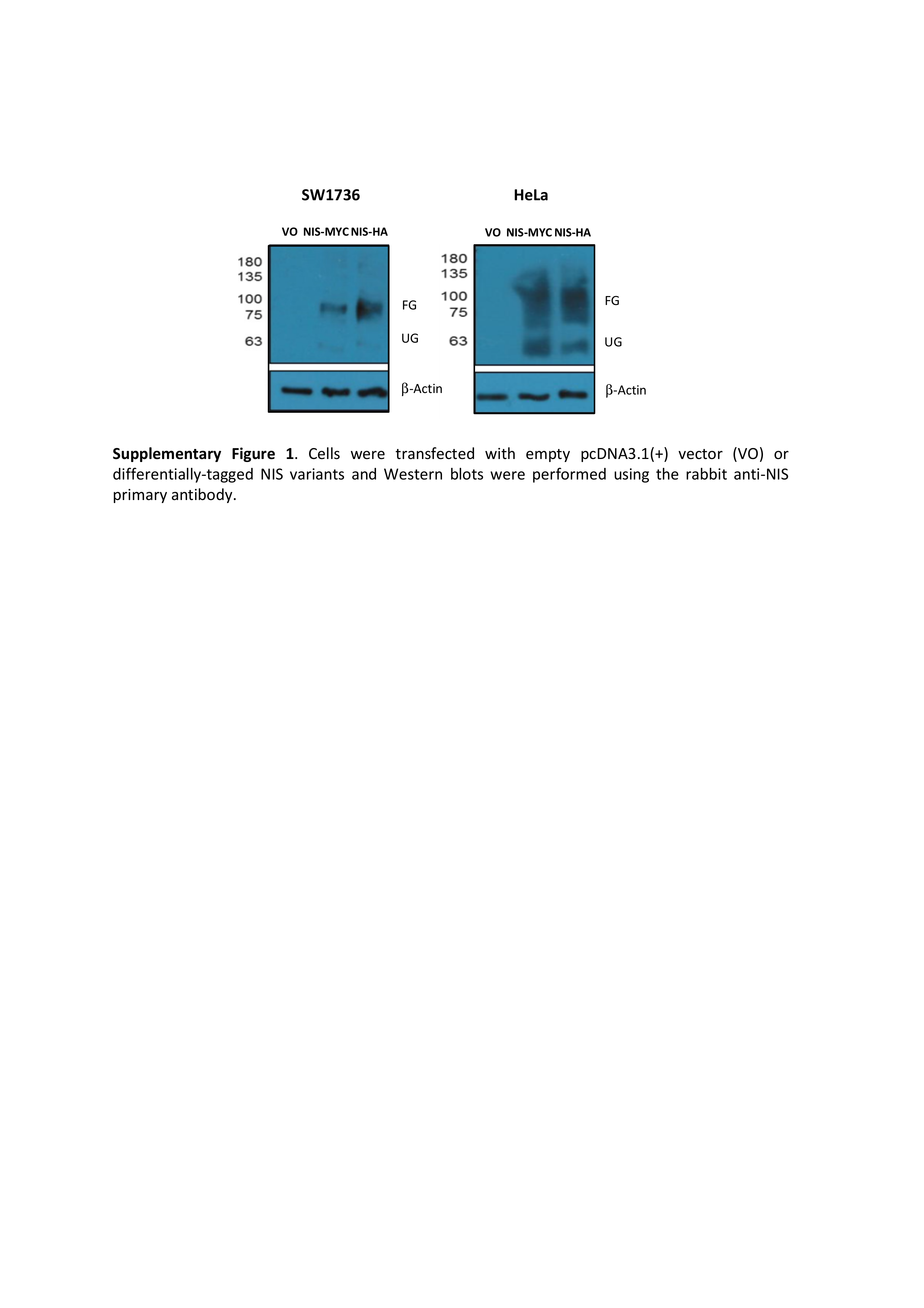

Supplement: Supplemental data [file Supp_Fig1.tif]

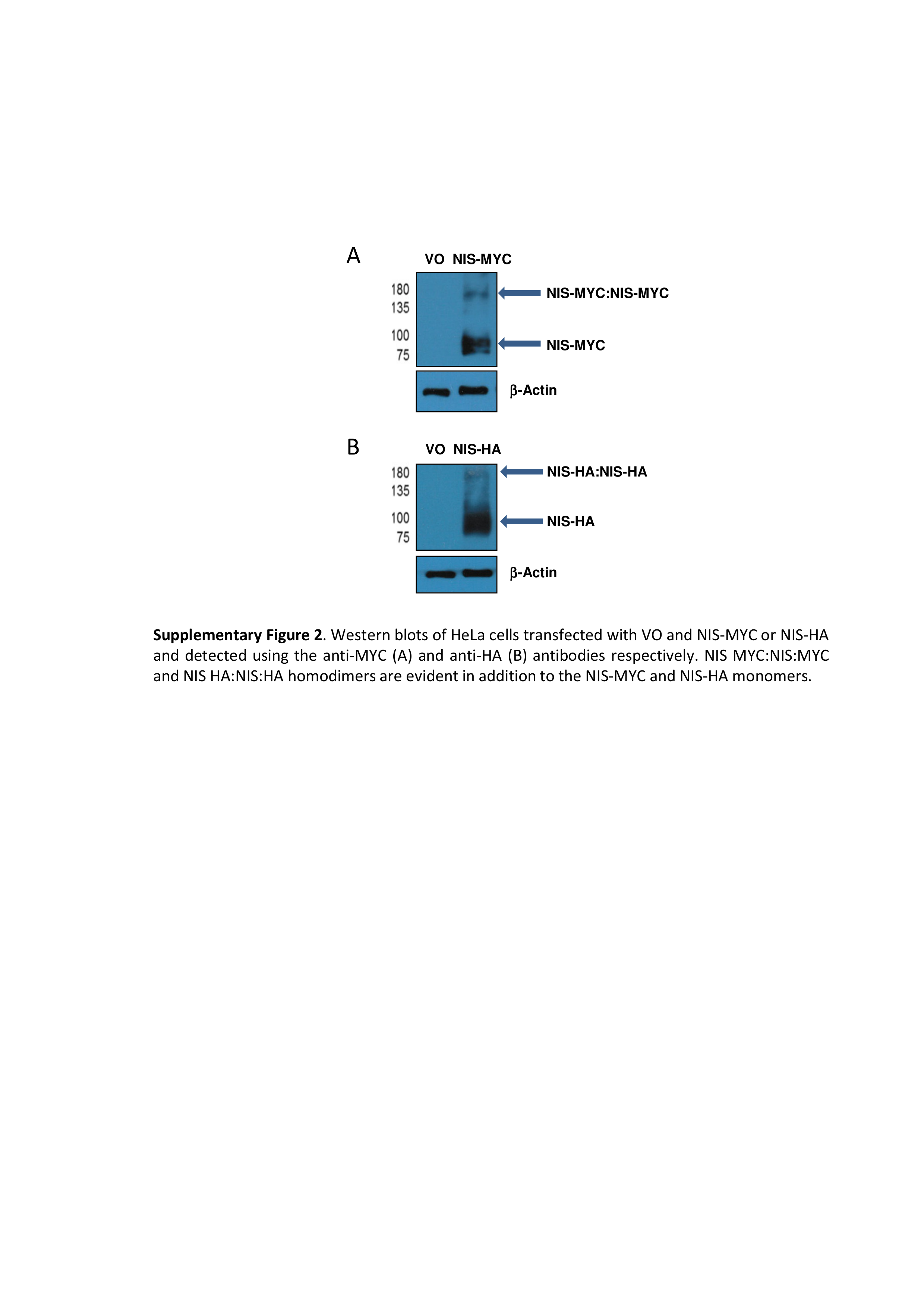

Supplement: Supplemental data [file Supp_Fig2.tif]

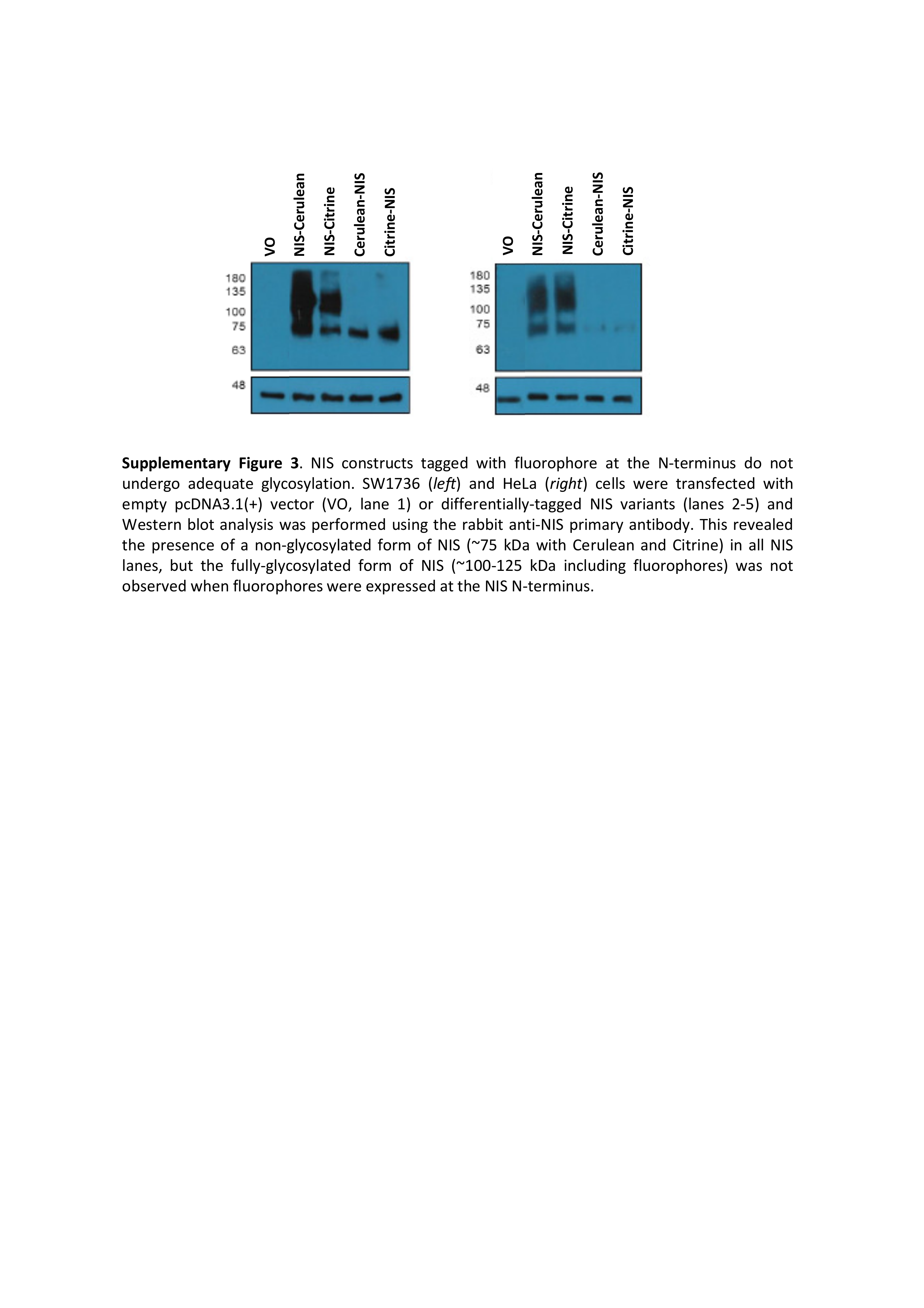

Supplement: Supplemental data [file Supp_Fig3.tif]

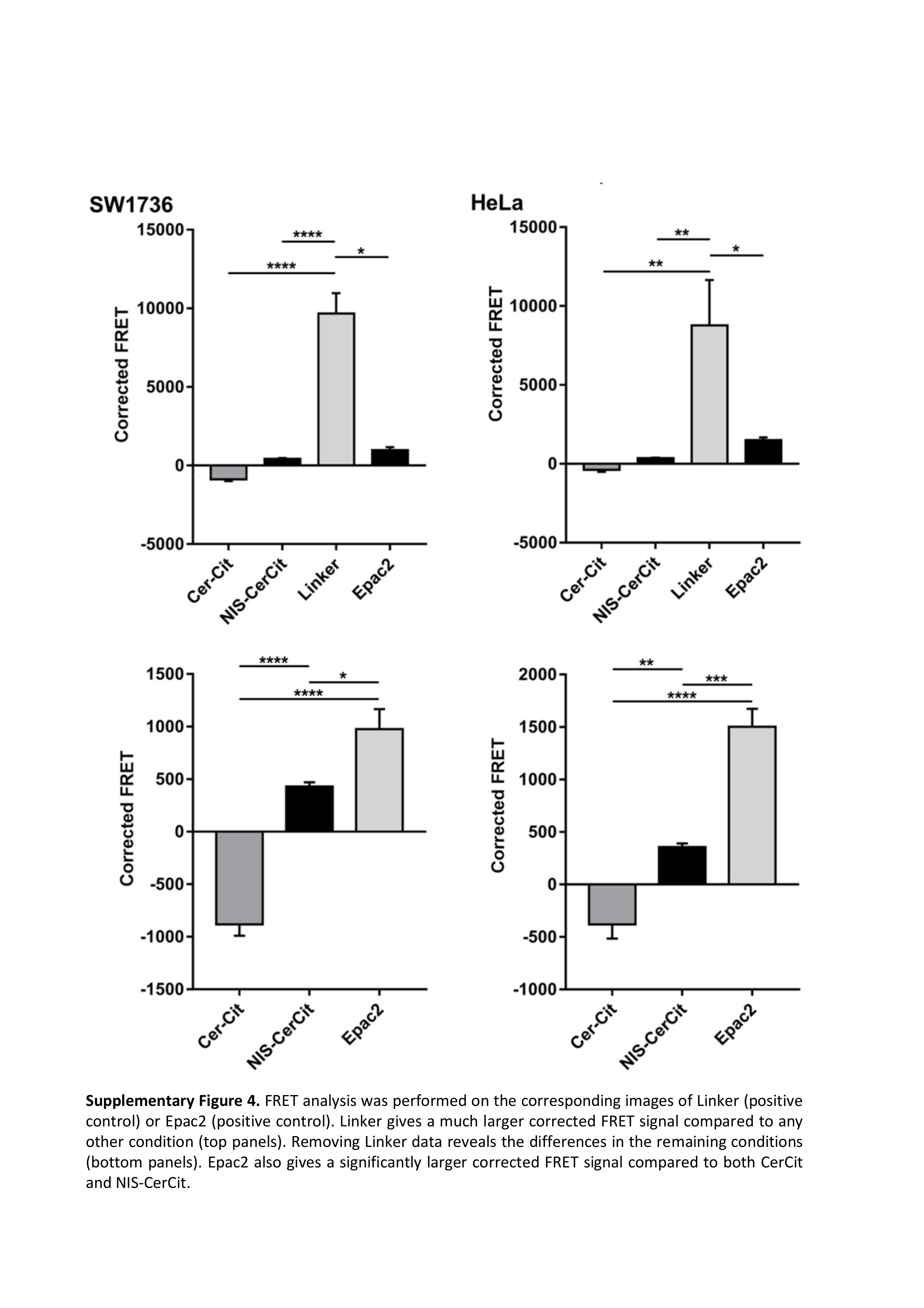

Supplement: Supplemental data [file Supp_Fig4.tif]

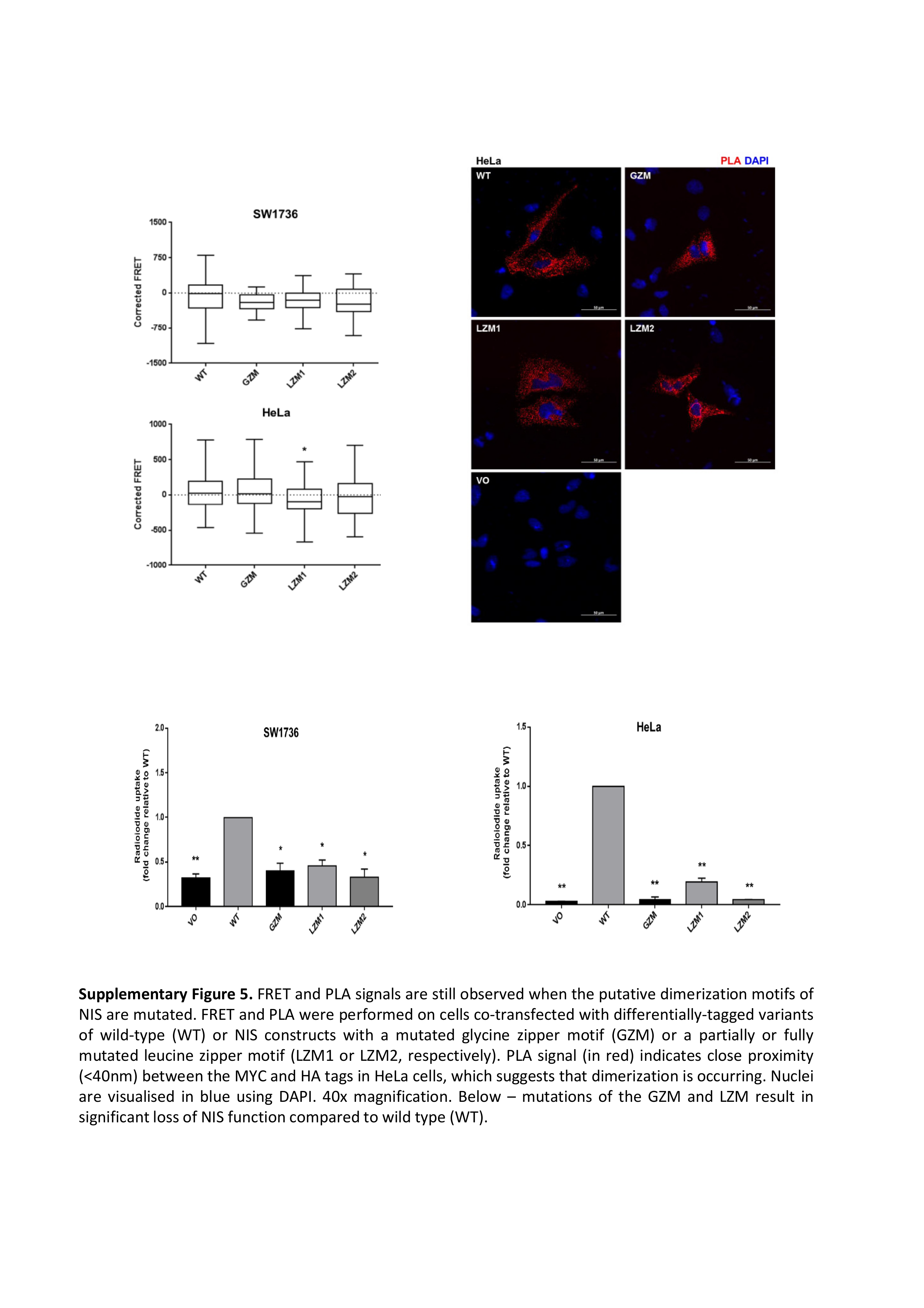

Supplement: Supplemental data [file Supp_Fig5.tif]

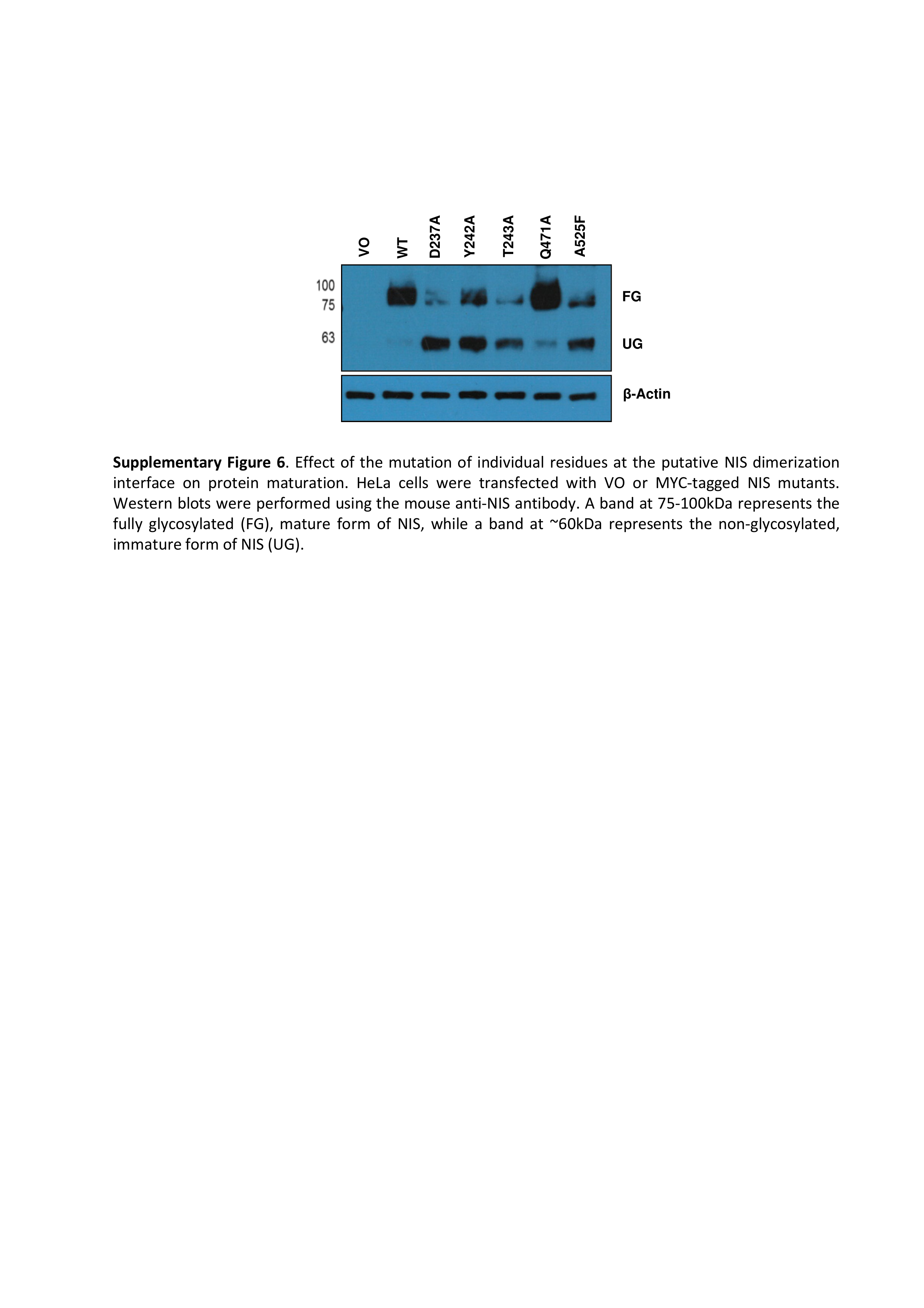

Supplement: Supplemental data [file Supp_Fig6.tif]

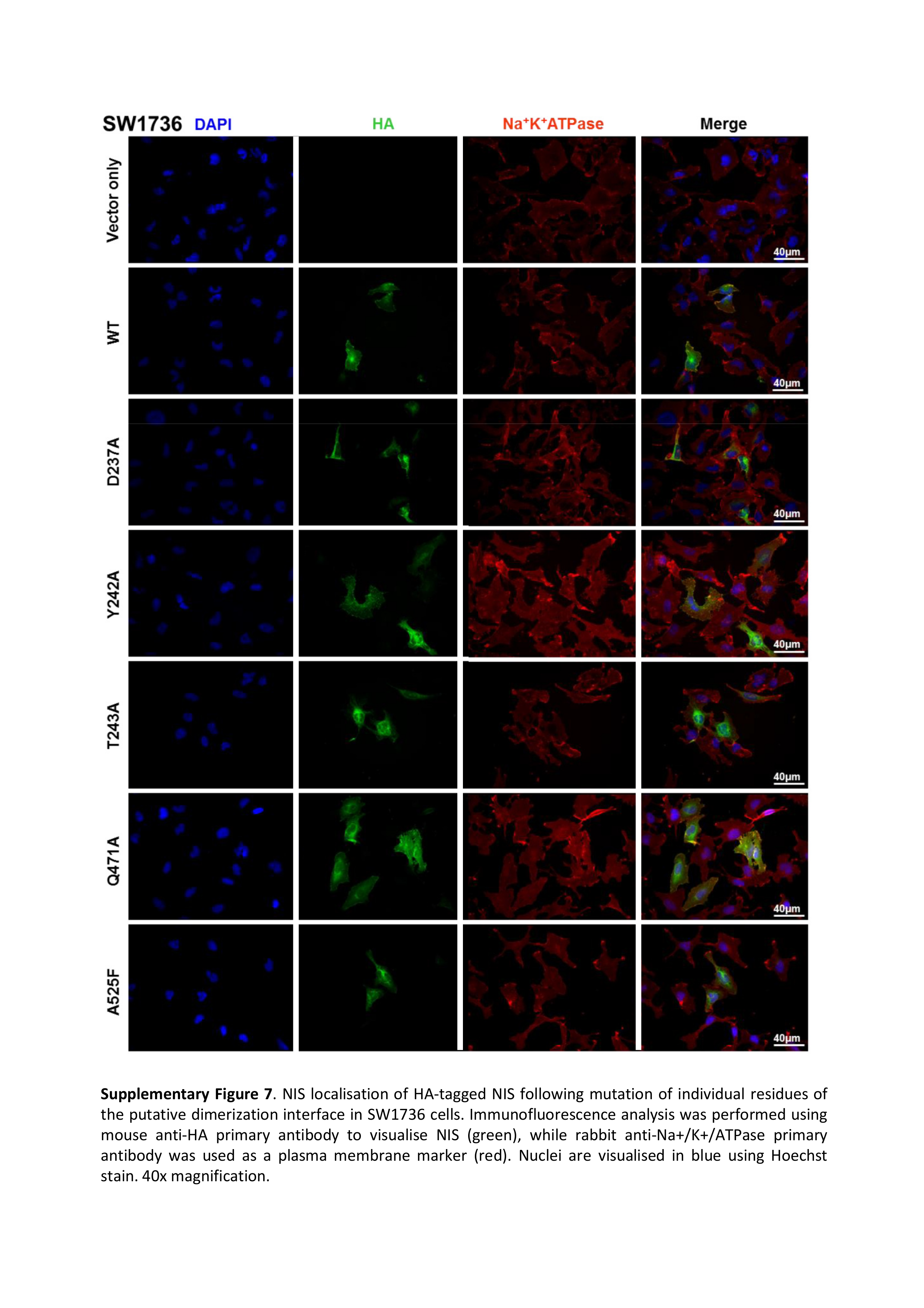

Supplement: Supplemental data [file Supp_Fig7.tif]

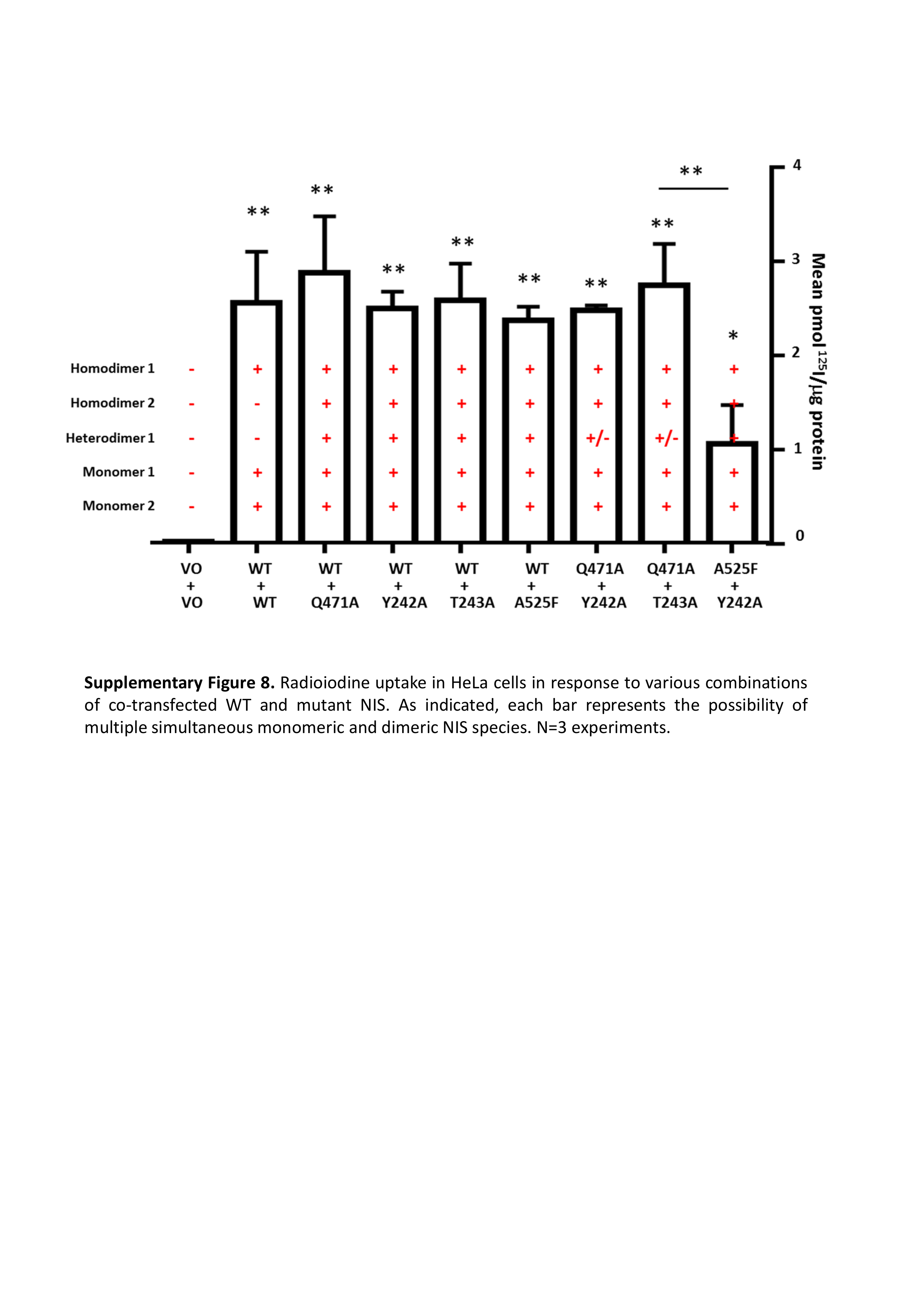

Supplement: Supplemental data [file Supp_Fig8.tif]
